# In Vitro Antibacterial Activity of Green Synthesized Silver Nanoparticles Using *Azadirachta indica* Aqueous Leaf Extract against MDR Pathogens

**DOI:** 10.3390/molecules27217244

**Published:** 2022-10-25

**Authors:** Omaish Alqahtani, Kiran K. Mirajkar, Anil Kumar K R., Mater H. Mahnashi, Ibrahim Ahmed Shaikh, Susweta Mitra, Harshitha Nagaraj, Sunil S. More, Uday M. Muddapur, Aejaz Abdullatif Khan, P. Renuka Sudarshan

**Affiliations:** 1 Department of Pharmacognosy, College of Pharmacy, Najran University, Najran 66462, Saudi Arabia; 2 Department of Biochemistry, University of Agricultural Sciences, Dharwad 580005, India; 3 Department of Pharmaceutical Chemistry, College of Pharmacy, Najran University, Najran 66462, Saudi Arabia; 4 Department of Pharmacology, College of Pharmacy, Najran University, Najran 66462, Saudi Arabia; 5 School of Basic and Applied Sciences, Dayananda Sagar University, Bangalore 560111, India; 6 Department of Biotechnology, KLE Technological University, Vidyanagar, Hubli 580031, India; 7 Department of General Science, Ibn Sina National College for Medical Studies, Jeddah 21418, Saudi Arabia

**Keywords:** *Magnaporthe oryzae*, MDR pathogens, *A. indica* silver nanoparticles, antibacterial activity, *Azadirachta indica*

## Abstract

Rice is the most important staple food crop feeding more than 50% of the world’s population. Rice blast is the most devastating fungal disease, caused by *Magnaporthe oryzae (M. oryzae*) which is widespread in rice growing fields causing a significant reduction in the yield. The present study was initiated to evaluate the effect of green synthesized silver nanoparticles (AgNPs) on the biochemical constituents of rice plants infected with blast. AgNPs were synthesized by using *Azadirachta indica* leaf extract and their characterization was performed using UV-visible spectroscopy, particle size analyser (PSA), scanning electron microscope (SEM), and X-ray diffraction (XRD) which confirmed the presence of crystalline, spherical shaped silver nanoparticles with an average size of 58.9 nm. After 45 days of sowing, artificial inoculation of rice blast disease was performed. After the onset of disease symptoms, the plants were treated with AgNPs with different concentrations. Application of nanoparticles elevated the activity of antioxidative enzymes such as superoxide dismutase, catalase, peroxidase, glutathione reductase, and phenylalanine ammonia-lyase compared to control plants, and total phenol and reducing sugars were also elevated. The outcome of this study showed that an increase in all biochemical constituents was recorded for *A. indica* silver nanoparticles-treated plants. The highest values were recorded in 30 ppm and 50 ppm AgNPs-treated plants, which showed the highest resistance towards the pathogen. Green synthesized AgNPs can be used in future for disease control in susceptible varieties of rice. The synthesized AgNPs using *A. indica* leaf extract have shown promising antibacterial activity when tested against 14 multidrug-resistant (MDR) bacteria comprising Gram-negative bacteria *Escherichia coli* (n = 6) and *Klebsiella pneumoniae* (n = 7) with a good zone of inhibition diameter, tested with the disc diffusion method. Based on these findings, it appears that *A. indica* AgNPs have promise as an antibacterial agent effective against MDR pathogens.

## 1. Introduction

The ‘green’ environment-friendly processes in agriculture and chemical technologies are becoming increasingly popular and are much needed because of worldwide problems associated with environmental concerns [1]. Silver is one of the most commercialized nanomaterials with five hundred tons of silver nanoparticles produced annually and estimated to increase in the next few years [2]. In recent years, noble metal nanoparticles have been the subject of focused research due to their unique, optical, electronic, mechanical, magnetic, and chemical properties that are significantly different from those of bulk materials [3]. Silver has been most extensively studied and used since ancient times to fight infections and prevent spoilage among the different antimicrobial agents. The antibacterial, antifungal, and antiviral properties of silver ions, silver compounds, and silver nanoparticles have been extensively studied. Silver is also found to be non-toxic to humans in minute concentrations. Microorganisms are unlikely to develop resistance against silver as compared to antibiotics as silver attacks a broad range of targets in the microbes [4]. Now, this technology is also being introduced in agriculture to control disease and pests in an effective way. Salve et al., synthesized nanoparticles using *M. longifolia*, evaluated for antimicrobial activity against human pathogenic microorganisms, anticancer activity, antioxidation, and anti-inflammatory activity [5]. For bovine mastitis, Taifa et al. developed a non-antibiotic, alternative, and economically viable treatment using copper nanoparticles [6].

Rice production is being continually threatened by diseases, insects, fungi, bacteria, viruses, and other stresses. Rice blast is one of the most destructive fungal diseases, which is widespread in rice-growing fields, causing a significant reduction in grain quality and yield. The blast causes huge losses ranging from 30% to 100% in all parts of the world where rice is being cultivated. Rice is a staple food for hundreds of millions of people around the world. Starvation is a very real possibility if crops fail for some reason, especially in poor and developing nations. Lesions form on the leaves, stems, peduncles, panicles, seeds, and even roots of rice plants when the fungus that causes rice blast infects them. This disease is considered to be one of the most critical plant diseases because of the severe damage it can do to crops [7]. Thus, it is paramount to find natural alternatives in the management and control of such crop diseases [8]. *Azadirachta indica* is a well-known pesticide and azadirachtin and salannin are the major components of neem oil with insecticidal properties. Hence, in the present study, an attempt was made to investigate the possible effects of *A. indica* AgNPs on plant growth and metabolism in rice crops infected with rice blast disease. Silver nanoparticles were synthesized through a green synthesis approach using *A. indica* leaves extract and characterized through scanning electron microscopy (SEM), particle size analyser (PSA), UV-visible spectroscope, and X-ray diffraction analyses. The study also included estimation of the biochemical parameters including total soluble sugar, total phenol contents, and the activities of antioxidant enzymes such as superoxide dismutase (SOD), catalase (CAT), peroxidase (POX), glutathione reductase (GR), and phytochemical precursor enzyme phenylalanine ammonia lyase (PAL) in AgNPs-treated plants.

## 2. Result and Discussion

### 2.1. Green Synthesis of Silver Nanoparticles from A. indica Leaf Extract

For the green synthesis of AgNPs, 2 mM of AgNO_3_ solution was prepared, and 10% of leaf extract was mixed in a 3:1 ratio respectively. Then the solution was kept in the dark at room temperature for about 24 h. The colour of the solution changed from brown to dark brown, which conformed to the synthesis of AgNPs.

### 2.2. Characterization of Green Synthesized AgNPs

The green synthesis of AgNPs using *A. indica* leaf extract was characterized by using various analytical techniques, such as UV-visible spectrophotometer (UV-Vis), particle size analyser (PSA), scanning electron microscope (SEM), and X-Ray diffraction. Details of the characterization AgNPs are explained in the following paragraphs.

#### 2.2.1. UV-Visible Absorption Spectrum of Green Synthesized AgNPs

**Figure 1:**
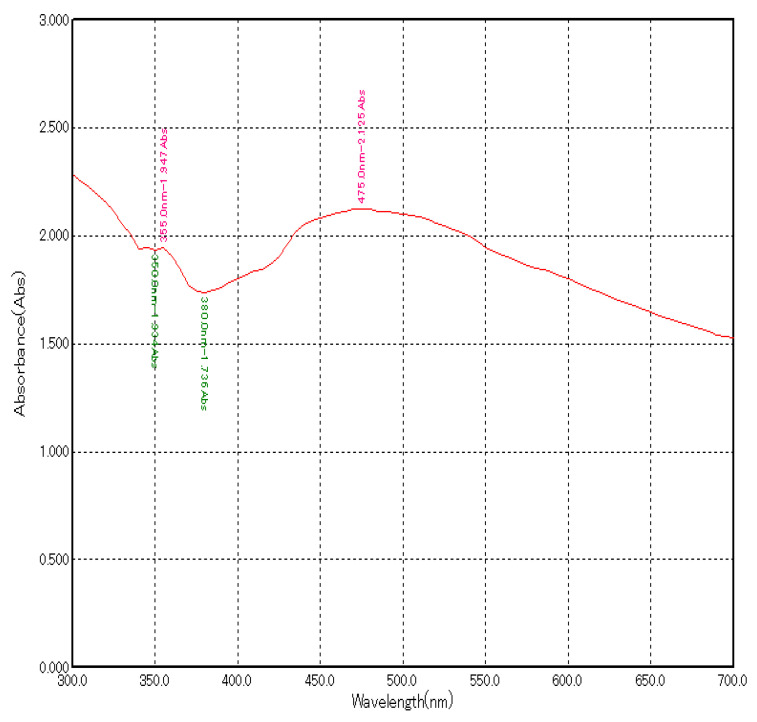
UV–Visible absorption spectrum of green synthesized AgNPs using *A. indica* leaf extract.

The addition of plant extract to the silver nitrate solution led to the change in colour from brown to dark brown indicating the formation of AgNPs due to the reduction of AgNO_3_ by the reducing agents present in the plant extract. Further confirmation of AgNP formation was obtained using a UV-visible spectrophotometer. Maximum absorbance of about 2.125 at 475 nm was observed (Figure 1).

#### 2.2.2. Particle Size Distribution Study by Particle Size Analyser (PSA)

**Figure 2:**
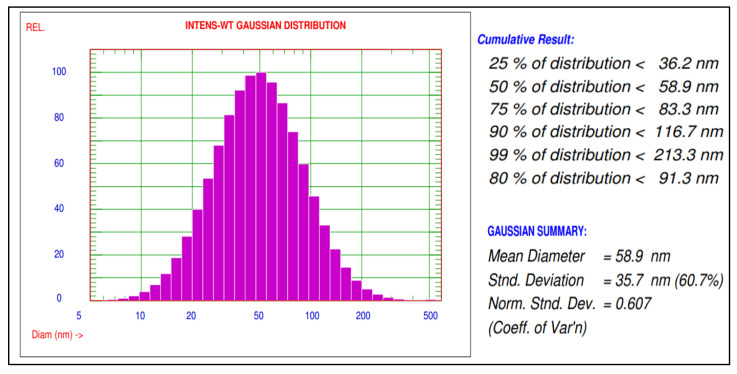
Particle size distribution of green synthesized AgNPs using *A. indica* leaf extract.

The mean diameter and particle size distribution of green silver nanoparticles synthesized using *A. indica* leaf extract were characterized using a particle size analyser (Nicomp NANOZ Z3000 PSS). The results of synthesized AgNPs have shown mean diameters of 80.3 nm and 58.9 nm before and after sonication, respectively, and the distribution of 90% AgNPs with a size smaller than 116.7 nm was recorded. These AgNPs were used to study the biochemical changes in rice plants infected with blast disease (Figure 2).

#### 2.2.3. SEM Micrographs of Green Synthesized AgNPs

**Figure 3:**
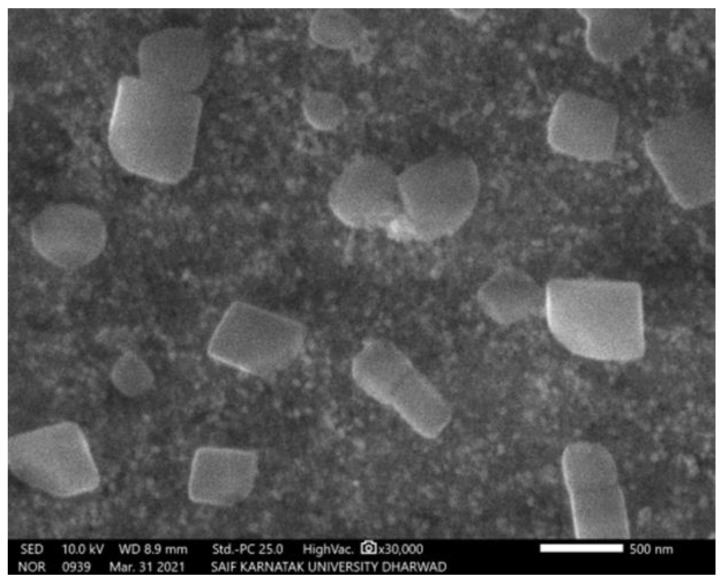
SEM micrograph of green synthesized AgNPs using *A. indica* leaf.

Scanning electron microscopy imaging was carried out to view the morphology and size of the silver nanoparticles. The SEM micrograph obtained for AgNPs showed high-density nanoscale particles with crystalline and spherical shapes (Figure 3). 

#### 2.2.4. XRD Spectrum Obtained for *A. indica* Leaf Extract-Mediated AgNPs

**Figure 4:**
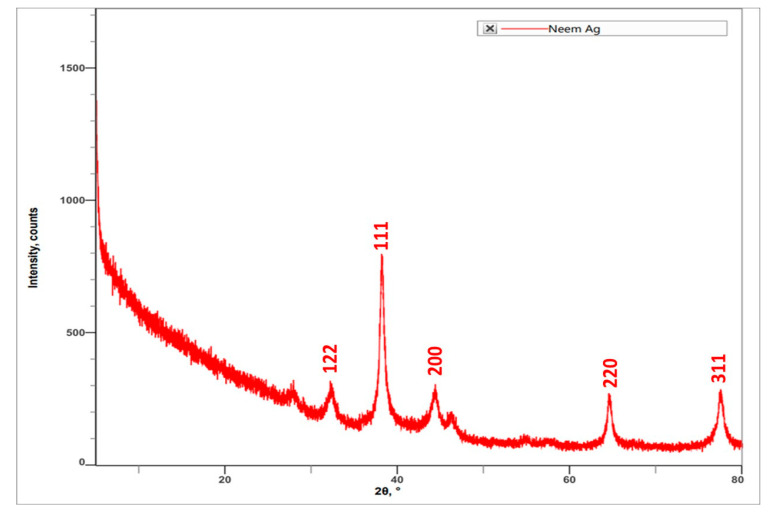
XRD spectrum obtained for *A. indica* leaf extract-mediated synthesized AgNPs.

Characterization of AgNPs synthesized by using *A. indica* leaf extract was carried out using XRD. The results indicated the peak angle of 2θ at 32.27°, 38.13°, 44.35°, 54.85°, 57.39°, 64.49°, 76.86°, and 77.40°. The highest intensity counts were at the angle of 32.27°, followed by 64.49°, 77.40°, 44.35°, 32.27°, 76.86°, 54.85°, and 57.39° (Figure 4). When compared with JCPDS No. 87-0579 standard, the obtained XRD spectrum confirmed that the synthesized silver nanoparticles were in crystalline nature. The peaks can be assigned to the planes (122), (111), (200), (220), and (311) facets of silver crystals.

### 2.3. To Study the Impact of Silver Nanoparticles on Physiological and Biochemical Changes in Rice Infected with M. oryzae

#### 2.3.1. Superoxide Dismutase (SOD)

**Figure 5:**
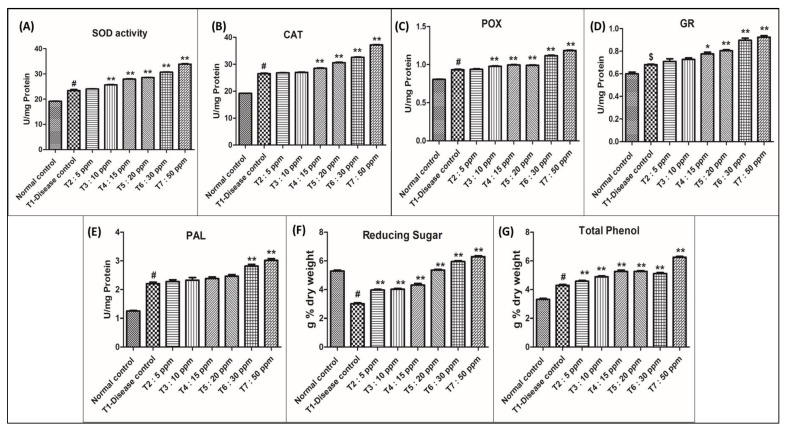
Graphical presentation for the biochemical parameters analysed in the study. Control: Rice plants not infected with *M. oryzae.* T_1_–T_7_ Rice plants infected with *M. oryzae*. Data are shown as mean ± SE (n = 3) at *p* < 0.05. One-way ANOVA followed by Tukey’s post-hoc test to compare means. ^#^ *p* < 0.001, ^$^ *p* < 0.05 compared to normal control group; * *p* < 0.01, ** *p* < 0.001 compared to disease control group.

The activity of Superoxide dismutase (Table 1 and Figure 5A) increased in silver nanoparticles-treated rice crops compared to that in the diseased and control groups.

When compared with control plants, superoxide dismutase activity increased by nearly 19.80% in the diseased plants (T1). All AgNPs-treated plants showed elevation in superoxide dismutase activity when compared with T1:0 ppm AgNPs. At 30 ppm: 30.69 U/mg protein (29%) and 50 ppm: 34.02 U/mg protein (43%), the nanoparticle-treated plants showed a significant elevation in enzyme activity and a reduction in disease severity. In T2, T3, T4, and T5 treatments the crop showed an elevation in enzyme activity but no significant reduction in disease severity. At 05 ppm: 1.26%, 10 ppm: 7.61%, 15 ppm: 17.11%, and 20 ppm: 20.18% elevation in enzyme activity was recorded.

#### 2.3.2. Catalase (CAT)

The activity of catalase (Table 2 and Figure 5B) increased in silver nanoparticles-treated rice crop compared to that of diseased and control groups.

When compared with control plants, catalase activity increased by nearly 27.90% in the diseased plants (T1). All AgNPs-treated plants showed elevation in catalase activity when compared with T1 (0 ppm AgNPs). At 30 ppm: 32.60 U/mg protein (22.42%) and 50 ppm: 37.20 U/mg protein (39.69%), the nanoparticle-treated plants showed significant elevation in enzyme activity and a reduction in disease severity. In T2, T3, T4, and T5 the crop showed elevation in enzyme activity but no significant reduction in disease severity at 05 ppm: 0.94%, 10 ppm: 1.46%, 15 ppm: 7.25%, and 20 ppm: 15.02% elevation in enzyme activity was recorded.

#### 2.3.3. Peroxidase (POX)

The activity of peroxidase (Table 3 and Figure 5C) increased in the silver nanoparticles-treated rice crop compared to that of diseased and control groups.

When compared with control peroxidase activity increased by 13.93% in the diseased plants (T1). All AgNPs-treated plants showed elevation in peroxidase activity, when compared with T1 (0 ppm AgNPs). At 30 ppm: 1.11 U/mg protein (19.83%) and 50 ppm: 1.19 U/mg protein (39.69%), the nanoparticle-treated plant showed a significant elevation in enzyme activity and a reduction in disease severity. In T2, T3, T4, and T5 the crop showed an elevation in enzyme activity but no significant reduction in disease severity. At 05 ppm: 0.75%, 10 ppm: 4.93%, 15 ppm: 5.89%, 20 ppm: 6.32% elevation in enzyme activity was recorded.

#### 2.3.4. Glutathione Reductase (GR)

The activity of glutathione reductase (Table 4 and Figure 5D) increased in the silver nanoparticles-treated rice crop compared to that of diseased and control groups.

When compared with the control, glutathione reductase activity increased by nearly 8.96% in the diseased plants (T1). All AgNPs-treated plants showed elevation in glutathione reductase activity compared with T1 (0 ppm AgNPs) at 30 ppm: 0.89 U/mg protein (28.18%) and 50 ppm: 0.92 U/mg protein (37.27), the nanoparticle-treated plant showed significant elevation in enzyme activity and a reduction in disease severity. In T2, T3, T4, and T5 the crop showed elevation in enzyme activity but no significant reduction in disease severity. At 05 ppm: 3.64%, 10 ppm: 7.27%, 15 ppm: 8.18%, 20 ppm: 11.82% elevation in enzyme activity was recorded.

#### 2.3.5. Phenylalanine Ammonia-Lyase (PAL)

The activity of phenylalanine ammonia-lyase (Table 5 and Figure 5E) increased in silver nanoparticles-treated rice crop compared to that of diseased and control.

When compared with the control, phenylalanine ammonia-lyase activity increased by nearly 43.18% in the diseased plants T1. All AgNPs-treated plants showed elevation in phenylalanine ammonia-lyase activity in all different treatments. At 30 ppm: 2.82 U/mg protein (28.18%) and 50 ppm: 3.02 U/mg protein (37.27%), the nanoparticle-treated plant showed significant elevation in enzyme activity and a reduction in disease severity. In T2, T3, T4, and T5 the crop showed an elevation in enzyme activity but no significant reduction in disease severity. At 05 ppm: 3.64%, 10 ppm: 7.27%, 15 ppm: 8.81%, 20 ppm: 11.82% elevation in enzyme activity was recorded.

#### 2.3.6. Reducing Sugar

Reducing sugar content (Table 6 and Figure 5F) in the leaves of AgNPs-treated rice infected with blast showed significant variation concerning concentration. Levels of reducing sugar in all AgNPs-treated plants were slightly increased when compared to the diseased rice plant (3.01 g% dry weight).

Among the treatments, plants treated with a higher concentration of silver nanoparticles (30 ppm: 5.35 and 50 ppm: 6.29 g% dry weight) showed a phenomenal increase in reducing sugar content compared to a low concentration (5 ppm: 3.96, 10 ppm: 4.02, 15 ppm: 4.31 and 20 ppm: 5.35 g% dry weight) and diseased (3.01 g% dry weight). 

Disease-inoculated rice plant leaves recorded a reduction in reducing sugar content (3.01 g% dry weight) as compared to control leaves (5.29 g% dry weight).

#### 2.3.7. Total Phenol

Total phenol content (Table 7 and Figure 5G) in the leaves of silver nanoparticle-treated rice infected with blast showed significant variation concerning concentration. Levels of total phenol in all nano-treated plants showed a slight increase when compared to the diseased rice plant (4.29 g% dry weight).

Among the treatments, plants treated with a higher concentration of silver nanoparticles (30 ppm: 5.12, 50 ppm: 6.25 g% dry weight) showed a phenomenal increase in total phenol content compared to low concentrations (05 ppm: 4.58, 10 ppm: 4.89, 15 ppm: 5.23 and 20 ppm: 5.26 g% dry weight) and diseased T1 (4.29 g% dry weight). 

Disease-inoculated rice plant leaves recorded total phenol content (3.32 g% dry weight) as compared to control leaves (4.29 g% dry weight). All the results of the biochemical analysis have been summarized in Figure 5.

#### 2.3.8. Antibacterial Studies against MDRs

**Figure 6:**
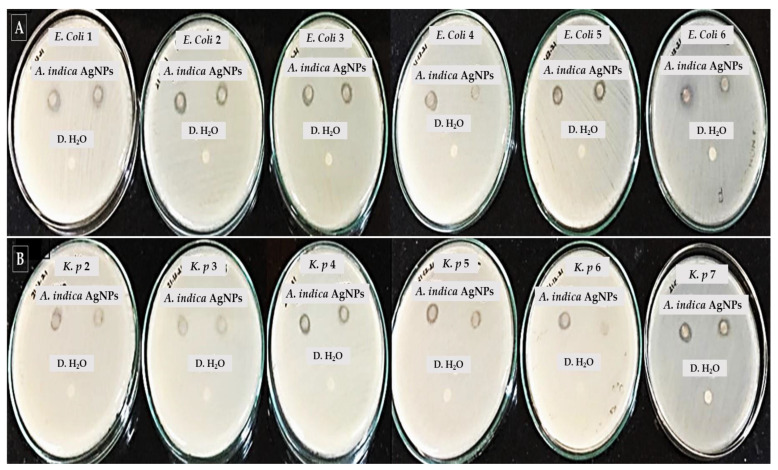
Zone of inhibition produced by *A. indica* AgNPs against clinical MDR pathogens: (**A**): *E. coli* strains (**B**) *K. pneumoniae* (*K. p*) strains. The distilled water (D. H_2_O) served as a control and a 100 µg/mL concentration of *A. indica* AgNPs was used to assess the antimicrobial activity against the MDR pathogens.

The AgNPs showed antibacterial activity against all the MDR pathogens included in this study. The results of disc diffusion assay are summarized in Table 8. The clear visible zone of inhibition exhibited by green synthesized AgNPs against the gram negative and gram positive MDR pathogens is depicted in Figure 6.

Nanotechnology is an emerging field with a vast range of applications in science and technology. The phytosynthesis technique makes use of eco-friendly, non-toxic, and safe materials like bacteria, fungi, plants, and plant extracts as alternatives to chemical and physical methods [3,9]. Previous studies have shown changes in biochemical and physiological factors in plants caused by the application of AgNPs. In vitro studies have shown that silver nanoparticles have antimicrobial activity. The usage of nanoparticles in the agriculture field is limited. In this context, the present study aims to synthesize AgNPs by using the *A. indica* leaf extract and provide their characterization, and their effect on biochemical changes in rice plants infected with blast (*M. oryzae*). The results of the present study are discussed under the following headings:

### 2.4. Green Synthesis of AgNPs Using A. indicia and Their Characterization

The present study reveals the successful synthesis of AgNPs from the neem leaf aqueous extract and their characterization. The green synthesis of AgNPs using different plant parts has been well documented in the literature [9,10,11,12]. In this study, the *A. indica* leaf was selected for the synthesis of AgNPs, because the leaf extract can reduce the AgNO_3_ solution to AgNPs due to the amines and alkaloids present in the leaves which also act as capping agents [10]. 

Green synthesized AgNPs were characterized for mean size, distribution, shape, morphology, and topography. In this study, green synthesized AgNPs were initially identified by the change in colour from light brown to a blackish colour after incubation, indicating the formation of AgNPs. Previous studies have obtained similar changes from colourless to brown-grey upon the addition of plant extract to AgNO_3_ solution as AgNPs were formed [13,14].

Characterization of AgNPs was performed using a UV-spectrophotometer by surface plasmon resonance and it showed a peak at 475 nm with an absorbance of 2.125. In various studies, peaks between 450–500 nm confirmed of synthesis of AgNPs [15,16,17]. 

A particle size analyzer (PSA) indicated a mean diameter of 58.9 nm and size distribution of AgNPs about 90% of is less than 116.7 nm size. Mohammad et al. and Prabha et al. [9,17] also observed a mean diameter of AgNPs of about 11.4 and 110.8 nm as results in their studies.

SEM and XRD results confirmed the presence of silver nanoparticles. SEM micrographs showed the spherical shape of AgNPs and the presence of silver nanoparticles in crystalline structure through the XRD spectrum [18]. SEM micrographs revealed that phytosynthesised AgNPs using *Catharanthus roseus* leaf extract were bunch form in shape.

### 2.5. To Study the Impact of AgNPs on Physiological and Biochemical Changes in Rice Infected with M. oryzae

#### 2.5.1. Superoxide Dismutase (SOD)

SOD catalyses the dismutation of superoxide radicals to hydrogen peroxide and oxygen and constitutes the most important enzyme in cellular defence because its activation directly modulates the amount of O_2_ and H_2_O_2_ [19]. SOD activity may reduce the risk of O_2_-radical formation. SODs are a group of metal-containing enzymes classified into three types according to their metal co-factor requirements viz., iron SOD (Fe-SOD) localized in the chloroplast, copper-zinc SOD (Cu/Zn-SOD) localized in the chloroplast, cytosol and the extracellular space and manganese SOD (Mn-SOD) is found mainly in mitochondria and peroxisomes [20]. 

In this study, biochemical changes in AgNPs-treated rice plants infected with blast disease were studied. The rice plant showed increased activity of SOD in all AgNPs-treated plants when compared with the control plants. As per the results obtained (Table 1), all the silver nanoparticle-treated rice plants showed an elevation in SOD. But only 30 ppm and 50 ppm silver nanoparticle-treated plants showed a reduction in blast disease severity along with an elevation in SOD activity.

Similar results were observed by Krishnaraj et al. (2012) [21] who studied the effect of AgNP application on *B. monnieri* biochemical activity. In their study, plants showed an elevation in SOD activity at 100 ppm AgNPs treatment. Similar results were obtained by Upadhyaya et al. (2017) [22] in their studies of zinc nanoparticles’ effect on rice seedlings.

#### 2.5.2. Catalase (CAT)

The plant possesses several antioxidant enzymes that eliminate (ROS) reactive oxygen species. Catalase (CAT) enzymes have a defensive role against ROS. It is responsible for the removal of toxic H_2_O_2_ in the cells, thereby protecting the cells from getting damaged. Up to a certain level, ROS production under stress may work as a signal for triggering defence responses via transduction pathways [23,24]. A high amount of ROS production in the cell causes cell death.

In this study, biochemical changes in AgNPs-treated rice infected with blast disease were analysed. The rice plants showed elevation in the catalase activity in all AgNPs-treated plants when compared with the control. As per the results obtained (Table 2), all the AgNPs-treated rice crops showed elevation in catalase, but crops treated with a higher concentration of AgNPs (30 ppm and 50 ppm) showed a highly significant reduction in blast disease severity along with elevated CAT activity.

Similar results were obtained by Guptha et al., 2018 [25] in their study on rice seedlings where they reported an increase in CAT activity with an increase in the concentration of AgNPs treatment.

#### 2.5.3. Peroxidase (POX) 

POX is a heme-containing protein that preferably oxidizes aromatic electron donors such as guaiacol and pyrogallol at the expense of H_2_O_2_. POX catalyses the conversion of cinnamyl alcohol to lignin by oxidative polymerization [26]. Vascular plants possess several gene-encoding numbers of POXs, and there might be some distinct physiological functions for each class in protecting the cell membrane against oxidative damage [27]. 

In the present study, biochemical changes in AgNPs-treated rice plants infected with blast disease were studied. The rice plant showed elevation in the peroxidase activity in all AgNPs-treated plants compared with the control. As per the results obtained (Table 3), all the AgNPs-treated rice plants showed elevation in peroxidase, but at the concentration of 30 ppm and 50 ppm, showed a reduction in blast severity along with highly significant elevation in peroxidase activity. The present study indicates more resistance towards disease at 30 and 50 ppm AgNPs, but all treatments showed elevation in all plant biochemical constituents.

The current study results were in line with an earlier study [25], where the POX activity increased with the increase in the concentration of AgNPs applied to on rice. Some studies reported that POX showed antifungal activity against a variety of fungal species, including *Mycophaerella arachidicola*, *Trichosporium vesiculosum*, *Coprinus comatus*, *Macrophomina phaseolina*, *Fusariumexo sporium*, and *Botrytis cenere* [28,29]. Elevation of POX activity along with an increase in the concentration of AgNPs in tomato plants was also reported by Noori et al. (2020) [30].

#### 2.5.4. Glutathione Reductase

Another important pathway involved in the control of ROS levels in the plant tissue is the glutathione/ascorbate cycle in which glutathione reductase (GR) plays a major role [31]. GR is a flavoprotein oxidoreductase that catalyses the reduction of glutathione disulphide (GSSG) to the sulphydryl form GSH. This enzyme employs NADPH as a reductant. 

As per our results (Table 4), all AgNPs-treated rice plants showed elevation in GR activity, and a reduction in disease severity was observed only in 30 and 50 ppm AgNPs-treated plants. The rice plants showed elevation in the GR activity in all AgNPs-treated conditions when compared with the control condition.

In another study, a similar elevation in GR activity was observed in rice seedlings [25], where the activity increased with the increase in AgNPs concentration. A similar elevation in GR content was observed [32] in the study on silver nanoparticle-induced morphological, physiological, and biochemical changes in the wheat plant. Elevation in all antioxidative enzymes and phenol content was recorded. 

#### 2.5.5. Phenylalanine Ammonia-Lyase

Phenylalanine ammonia-lyase is the key enzyme in the plant phenylpropanoid pathway catalysing the synthesis of secondary metabolites from L-phenylalanine including lignin, flavonoid, and phytoalexins. PAL enzyme catalyses the nonoxidative deamination of L-phenylalanine to form trans-cinnamic acid and a free ammonium ion. The conversion of the amino acid phenylalanine to trans-cinnamic acid is the entry step for the channelling of carbon from primary metabolism into phenylpropanoid secondary metabolism in plants.

As per the results obtained (Table 5) among all the treatments T6: 30 ppm and T7: 50 ppm recorded the highest PAL activity. Whereas the lowest PAL activity was observed in the control rice plant. Blast-infected plants also showed a small elevation compared to the control plant in PAL activity. In the present study, biochemical changes in AgNPs-treated rice infected with blast disease were analysed. 

As per the results obtained (Table 5) all the AgNPs-treated rice plants showed elevation in PAL activity. At T6: 30 ppm and T7: 50 ppm AgNPs-treated rice plants showed a reduction in blast severity and elevation in PAL activity. The present study indicates that the crop showed more resistance towards disease at T6: 30 ppm and T7: 50 ppm AgNPs treatments. Similar results have been reported in earlier studies in different plants [33,34].

#### 2.5.6. Reducing Sugars

Sugars constitute the primary metabolite providing energy to the system and structural material for defence responses in plants, and act as signal molecules interacting with the plant immune system. Sugars cause oxidative burst at the early stages of any pathogen or pest infection, increasing the lignification of cell walls, stimulating the synthesis of flavonoids, and inducing certain pathogenesis-related proteins. Some sugars act as priming agents inducing higher plant resistance to pathogens, as reported [35].

In this study, the response of AgNPs applied to rice plants infected with blast was studied to correlate the role of reducing sugar content upon blast infection and NP application. As per the results obtained (Table 6), the control plant recorded the highest reducing sugar content, and the lowest reducing sugar content was observed in the blast-infected plant i.e., T1: 0 ppm AgNPs. Blast induced a decrease in sugar content. All other treatments showed elevation in reducing sugar content after the application of AgNPs.

Krishnaraj et al. (2012) [21] studied biologically synthesized AgNPs which exerted a slight stress on the growth and metabolism of *B. monnieri*. They also observed elevation in total sugar content after 5 mg/L concentration of AgNPs application.

#### 2.5.7. Total Phenol

Phenols play an important role in the plant defence system against insects, plant pathogens, and cyclic reduction of ROS such as superoxide anion and hydroxide radicals, H_2_O_2_, and singlet oxygen [36]. Phenols inhibit disease development through different mechanisms, such as inhibition of extracellular fungal enzymes (cellulase, pectinase, laccase, and xylanase), fungal oxidative phosphorylation, nutrition deprivation, and antioxidant activity in the plant tissue [37,38].

In this study, as per the results obtained (Table 7) among control and infected rice plants, the control plants showed a low amount of total phenol content. AgNPs-treated rice plants showed elevation in total phenol content relative to the concentration of AgNPs applied. 

Similar results were reported earlier [21], where the total phenol increased in *Baco pamonnieri* plant on the application of AgNPs.

## 3. Materials and Methods

### 3.1. Experimental Treatment Details

The different experimental groups used in the present study are shown in Table 9.

### 3.2. Green Synthesis and Characterization of AgNPs Using A. indica Leaf Extract

Fresh leaves of *A. indicia* were collected and were thoroughly washed several times with tap water followed by distilled water to remove the dust particles and air-dried at room temperature for three days.

### 3.3. Preparation of the A. indica Aqueous Leaf Extract

The aqueous leaf extract was prepared following the protocol described by Shukla et al., 2009, with minor modifications [10]. Ten grams of fresh leaves were ground using a pestle and mortar in 100 mL of distilled water (10% aqueous plant extract), filtered through Whatman filter paper No. 1, and the filtrate was stored in a refrigerator at 4 °C until further use.

### 3.4. Preparation of AgNO_3_ Solution

Silver nitrate was procured from the Sisco Research Laboratories Pvt. Ltd. (SRL, Mumbai, India). An amount of 2 mM silver nitrate solution was used for the synthesis of AgNPs.

### 3.5. Green Synthesis of AgNPs Using A. indica Aqueous Leaf Extract

AgNO_3_ was used as a precursor for the green synthesis of AgNPs, and the leaf extract served as both a reducing and a capping agent. As mentioned by Roy et al. (2017), 10 mL of leaf extract and 30 mL of AgNO_3_ (2 mM) were thoroughly combined and kept for reaction in the dark for approximately 24 h [39,40,41].

### 3.6. Characterization of Green Synthesized AgNPs

The synthesized nanoparticles were characterized in terms of shape, size, and morphology using a UV-Visible spectrophotometer (UV-Vis), PSA, SEM, and XRD. ELICO UV1900 Double Beam Spectro-photometer with wavelength scan in the range of 190 nm to 1100 nm and bandwidth of 2 nm was used to analyse the spectra of AgNPs. Between 200 and 700 nm in wavelength, green AgNPs were scanned. A PSA (Particle Size Analyzer) was used to characterize green synthesized AgNPs in terms of mean diameter and distribution (Nicomp, Nicomp NANOZ Z3000 PSS USA). SEM (Scanning Electron Microscopy-Carl Zeiss-EVO-18-UK) was employed to analyse the topology of AgNPs. The structural properties of green synthesized AgNPs were evaluated using an X-ray diffractometer (Powder) (Model: SmartLab SE) with fully automated alignment controlled by a computer, SAXS capabilities, and an optional D/teXUltra high-speed, position-sensitive detector system with 2D: 2° to 150°.

### 3.7. Plant Sample

Rice (*Oryza sativa* L.) INTAN variety (blast-susceptible) was chosen to study the biochemical response of antioxidant enzymes. Rice was evaluated by pot culture experiments in a polyhouse. Forty-five days after sowing, the artificial inoculation of *M. oryzae* pure culture was performed by spraying to ensure the maximum disease pressure. After the onset of disease symptoms, the AgNP treatment was given as per the treatment details; then, leaf samples were collected after 72 h of treatment for determining the reducing sugar, total phenol, and antioxidant enzyme activities such as superoxide dismutase (SOD), catalase (CAT), glutathione reductase (GR), peroxidase (POX), and phenylalanine ammonia-lyase (PAL).

### 3.8. Extraction of Enzyme and Determination of Protein Content

Fresh leaf tissues after collection were processed immediately for enzyme extraction between 0 °C and 4 °C and used for the assay. In order to measure the enzyme activities, 0.5 g of leaf tissues were taken and ground into a fine powder with liquid nitrogen, and extracted with 2 mL of 0.05 M sodium phosphate buffer of pH 7.8 and pH 7.0 for SOD and CAT, respectively. POX was extracted in 0.1 M potassium phosphate buffer (pH 7.0). Grinding buffer for GR included 0.1 M Tris–HCl pH 7.8 and 2 mM dithiothreitol (DTT). PAL was extracted in 0.1 M Tris buffer (pH 8.5). Then, the homogenate was centrifuged at 14,000 rpm for 20 min at 4 °C and the supernatant was used as an enzyme source for assay. Protein content was estimated using Lowry’s method.

### 3.9. Biochemical Assays

Antioxidant enzyme assays including superoxide dismutase, catalase, peroxidase, glutathione reductase, phenylalanine ammonia-lyase, and estimation of reducing sugar and total phenols were carried out using standard biochemical procedures [42,43,44,45,46,47,48,49,50].

#### 3.9.1. Superoxide Dismutase Activity

The activity of SOD (EC 1.15.1.1) was determined photochemically at 560 nm by the method of Beauchamp and Fridovich. An amount of 3 mL of reaction mixture contained 20 μL of SOD enzyme extract, p-nitrobluetetrazolium chloride (NBT (33 μM), L-methionine (10 mM), EDTA (0.66 μM)) in a 50 mM potassium phosphate buffer, pH 7.8 and riboflavin (3.3 μM). The reaction was initiated by adding riboflavin in a glass tube illuminated by a 15 W fluorescent lamp for 20 min at 25 °C. The increase in optical density of the blue formazan product formed by NBT photoreduction was recorded at 560 nm. A blank was used with all the above constituents but kept in the dark. One unit of SOD activity is defined as the amount of enzyme required to inhibit 50% of photo-reduction of NBT per minute, and specific activity is expressed as IUmg^−1^ protein.

#### 3.9.2. Catalase Activity

Catalase (EC 1.11.1.6) activity was determined by the Beers and Sizers spectrophotometric method. The assay mixture comprised 2.98 mL of 16.65 mM hydrogen peroxide in 50 mM phosphate buffer, pH 7.0 and 20 μL of enzyme source was used to start the reaction. The decrease in optical density at 240 nm was recorded for 5 min against the substrate blank. One unit of catalase is defined as the amount of enzyme that transforms one μmole of hydrogen peroxide per minute at pH 7.0 at 25 °C, and specific activity was expressed as μmol min^−1^ mg^−1^ protein.

#### 3.9.3. Peroxidase Activity

Peroxidase (EC 1.11.1.7) activity was spectrophotometrically assayed by the method of Chance and Maehly. The assay was initiated by the addition of 20 μL of the enzyme preparation to a reaction mixture comprising 2.88 mL of 100 mM potassium phosphate buffer, pH 7.0, 50 μL of 20 mM guaiacol, and 50 μL of 0.042% H_2_O_2_, and increase in optical density at 436 nm was monitored for 5 min. One POX unit is defined as the amount of enzyme which catalyses the production of one µmole of oxidized guaiacol per minute at 25 °C, and specific activity is expressed as μmole min^−1^ mg^−1^ protein.

#### 3.9.4. Glutathione Reductase Activity

Glutathione reductase (EC 1.8.1.7) activity was assayed by the spectrophotometric method of Mavis and Stellwagen [46]. The assay mixture comprised 100 μL of 30 mM oxidized glutathione (GSSG), 1.5 mL of 0.1 M potassium phosphate buffer with 0.0034 M EDTA, pH 7.6, 0.35 mL of 0.8 mM reduced ß-nicotinamide adenine dinucleotide phosphate (NADPH) and 0.95 mL of water. The decrease in optical density at 340 nm on the addition of 100 μL of the enzyme to the reaction mixture was recorded for 5 min. One GR unit is defined as the amount of enzyme required to oxidise 1.0 micromole of NADPH per minute at pH 7.6 and 25 °C, and specific activity is represented as a micromole per min per mg protein.

#### 3.9.5. Phenylalanine Ammonia Lyase Activity

Phenylalanine ammonia-lyase activity (EC.4.3.1.5.) was spectrophotometrically determined by the method of Paltonen and Karjalainen. The reaction mixture contained 50 μL of enzyme preparation and 2.5 mL of 0.2% L-phenylalanine in 0.1 M Tris buffer (pH 8.5) which was incubated for 1 h at 40 °C. The reaction was terminated by the addition of 0.5 mL of 0.2 M HCl, and the optical density was measured at 290 nm using substrate blank. One unit of PAL is defined as the amount of enzyme that forms 1 μmol of trans-cinnamate per minute from L-Phenylalanine, and the specific activity of PAL was expressed as μmol/min/mg protein.

#### 3.9.6. Estimation of Total Phenols

One gram of powdered, dried leaf tissue was extracted with10 mL of hot 80% ethyl alcohol [49]. The colorimetric method of Bray and Thorpe was employed for the estimation of total phenols [50]. The total phenol content was represented as mg per gram dry weight.

#### 3.9.7. Estimation of Reducing Sugars

One gram of powdered, dried leaf material was introduced in hot 80% ethyl alcohol for 15 min and then homogenized using a pestle and mortar. The obtained homogenate was filtered, and the leaf tissue was re-extracted two or three times. The filtrate was made up to a final volume of 10 mL with 80% ethanol. Reducing sugars were estimated by Nelson’s modification of Somogi’s method [47]. The reducing sugars were represented as mg per gram dry weight.

### 3.10. Bacterial Strains Used in This Study

We used 14 multi-drug resistant (MDR) bacteria comprising Gram-negative bacteria-*Escherichia coli* (n = 6) and *Klebsiella pneumoniae* (n = 7). These clinical strains were phenotypically and genotypically characterized in our previous study and found to possess a wide spectrum of beta-lactamase genes conferring antibiotic resistance [51,52]. ATCC 25922 *E. coli* was used as a quality control strain.

### 3.11. In Vitro Antibiotic Susceptibility Test of Green Synthesized AgNPs against MDR Bacteria

#### Disc Diffusion Method

The antibacterial activity of green synthesized AgNPs against the selected MDR bacterial (n = 13) strains was carried out using the Kirby–Bauer Disc Diffusion method [53]. The bacterial strains were grown until attaining the McFarland standard of O.D: 0.5. The inoculum was spread in Muller Hinton Agar (MHA) (Himedia, India) using sterile cotton swabs, and a sterile antimicrobial susceptibility disk was loaded on the plates with 10 μL of green synthesized AgNPs. The distilled water served as a control, and 100 µg/mL concentration of *A. indica* AgNPs was used to assess the antimicrobial activity against the MDR pathogens. The plates were incubated at 37 °C for 48 h and zone of inhibition was recorded.

### 3.12. Statistical Analysis

The data of the experiment will be analysed statistically by the following procedure described by Gomez and Gomez [54]. Data were analysed using GraphPad Prism version 6 (San Diego, CA, USA). The results are expressed as mean ± SD. The differences between various groups were calculated using a one-way analysis of variance (ANOVA) followed by the post-Tukey’s test. A *p*-value less than 0.05 was considered to be significant.

## 4. Conclusions

AgNPs synthesis was mediated by *A. indica* leaves extract and characterized using SEM, particle size analyser, UV-visible spectroscope, and X-ray diffraction analyses. The outcome of this study showed that an increase in all biochemical constituents such as reducing sugar, total phenol contents, and activities of antioxidant enzymes such as SOD, CAT, POX, GR, and phytochemical precursor enzyme PAL was observed in AgNPs-treated plants. The higher values were recorded in 30 ppm and 50 ppm AgNPs-treated plants and showed the highest resistance towards the pathogen. Green synthesized AgNPs can be used in future for disease control in susceptible varieties of rice and have shown promising antibacterial activity against hospital-borne MDR pathogenic strains of concern.

## Figures and Tables

**Table 1:** Activity of Superoxide dismutase enzyme in different nanoparticle treatments of the rice plant infected with blast.

Treatment	Activity of Superoxide Dismutase (U/mg Protein)	% Variation Compared with T_1_
Control	19.08	-
T_1_: 0 ppm AgNPs (DC)	23.79 ^#^	-
T_2_: 05 ppm AgNPs	24.09	1.26
T_3_: 10 ppm AgNPs	25.60 *	7.61
T_4_: 15 ppm AgNPs	27.86 *	17.11
T_5_: 20 ppm AgNPs	28.59 *	20.18
T_6_: 30 ppm AgNPs	30.69 *	29.00
T_7_: 50 ppm AgNPs	34.02 *	43.00
C.D.	2.836	
S.E(m)	0.938	

Control: Rice plants not infected with *M. oryzae*. T_1_–T_7_ Rice plants infected with *M. oryzae*. Data are demonstrated as mean ± SE (n = 3) at *p* < 0.05. One-way ANOVA followed by Tukey’s post-hoc test to compare means. ^#^ *p* < 0.001, compared to control group; * *p* < 0.001, compared to disease control group.

**Table 2:** Activity of catalase enzyme in different nanoparticle treatments of the rice plant infected with blast.

Treatment	Activity of Catalase (U/mg Protein)	% Variation Compared with T_1_
Control	19.2	-
T_1_: 0 ppm AgNPs (DC)	26.63 ^#^	-
T_2_: 05 ppm AgNPs	26.88	0.94
T_3_: 10 ppm AgNPs	27.02	1.46
T_4_: 15 ppm AgNPs	28.56 *	7.25
T_5_: 20 ppm AgNPs	30.63 *	15.02
T_6_: 30 ppm AgNPs	32.60 *	22.42
T_7_: 50 ppm AgNPs	37.20 *	39.69
C.D.	2.531	
S.E(m)	0.837	

Control: Rice plants not infected with *M. oryzae*. T_1_-T_7_ Rice plants infected with *M. oryzae*. Data are shown as mean ± SE (n = 3) at *p* < 0.05. One-way ANOVA followed by Tukey’s post-hoc test to compare means. ^#^ *p* < 0.001, compared to control group; * *p* < 0.001, compared to disease control group.

**Table 3:** Activity of peroxidase enzyme in different nanoparticle treatments of rice plant infected with blast.

Treatment	Activity of Peroxidase (U/mg Protein)	% Variation Compared with T_1_
Control	0.803	-
T_1_: 0 ppm AgNPs (DC)	0.933 ^#^	-
T_2_: 05 ppm AgNPs	0.940	0.75
T_3_: 10 ppm AgNPs	0.979 *	4.93
T_4_: 15 ppm AgNPs	0.988 *	5.89
T_5_: 20 ppm AgNPs	0.992 *	6.32
T_6_: 30 ppm AgNPs	1.118 *	19.83
T_7_: 50 ppm AgNPs	1.194 *	27.97
C.D.	0.087	
S.E(m)	0.029	

Control: Rice plants not infected with *M. oryzae*. T_1_–T_7_ Rice plants infected with *M. oryzae*. Data are demonstrated as mean ± SE (n = 3) at *p* < 0.05. One-way ANOVA followed by Tukey’s post-hoc test to compare means. ^#^ *p* < 0.001, compared to control group; * *p* < 0.001, compared to disease control group.

**Table 4:** Activity of glutathione reductase enzyme in different nanoparticle treatments of rice plant infected with blast.

Treatment	Activity of Glutathione Reductase (U/mg Protein)	% Variation Compared with T_1_
Control	0.60	-
T_1_: 0 ppm AgNPs (DC)	0.67 ^#^	-
T_2_: 05 ppm AgNPs	0.71	5.97
T_3_: 10 ppm AgNPs	0.73	8.96
T_4_: 15 ppm AgNPs	0.78 *	16.42
T_5_: 20 ppm AgNPs	0.80 **	19.40
T_6_: 30 ppm AgNPs	0.89 **	32.84
T_7_: 50 ppm AgNPs	0.92 **	37.31
S.E(m)	0.027	
C.D.	0.082	

Control: Rice plants not infected with *M. oryzae*. T_1_–T_7_ Rice plants infected with *M. oryzae.* Data are shown as mean ± SE (n = 3) at *p* < 0.05. One-way ANOVA followed by Tukey’s post-hoc test to compare means. ^#^ *p* < 0.05, compared to control group; * *p* < 0.01, ** *p* < 0.001 compared to disease control group.

**Table 5:** Activity of Phenylalanine ammonia-lyase enzyme in different nanoparticle treatments of the rice plant, infected with blast.

Treatment	Activity of Phenylalanine Ammonia-Lyase (U/mg Protein)	% Variation Compared with T_1_
Control	1.25	-
T_1_: 0 ppm AgNPs (DC)	2.20 ^#^	-
T_2_: 05 ppm AgNPs	2.28	3.64
T_3_: 10 ppm AgNPs	2.36	7.27
T_4_: 15 ppm AgNPs	2.38	8.18
T_5_: 20 ppm AgNPs	2.46	11.82
T_6_: 30 ppm AgNPs	2.82 *	28.18
T_7_: 50 ppm AgNPs	3.02 *	37.27
S.E(m)	0.097	
C.D.	0.294	

Control: Rice plants not infected with *M. oryzae*. T_1_–T_7_ Rice plants infected with *M. oryzae*. Data are shown as mean ± SE (n = 3) at *p* < 0.05. One-way ANOVA followed by Tukey’s post-hoc test to compare means. ^#^ *p* < 0.001, compared to control group; * *p* < 0.001 compared to disease control group.

**Table 6:** Estimation of reducing sugar in different nanoparticle treatments of rice plant infected with blast.

Treatment	Reducing Sugar (g% Dry Weight)	% Variation Compared with T_1_
Control	5.29	-
T_1_: 0 ppm AgNPs (DC)	3.01 ^#^	-
T_2_: 05 ppm AgNPs	3.96 *	31.56
T_3_: 10 ppm AgNPs	4.02 *	33.55
T_4_: 15 ppm AgNPs	4.31 *	43.19
T_5_: 20 ppm AgNPs	5.35 *	77.74
T_6_: 30 ppm AgNPs	5.95 *	97.67
T_7_: 50 ppm AgNPs	6.29 *	108.97
S.E(m)	0.225	
C.D.	0.682	

Control: Rice plants not infected with *M. oryzae*. T_1_–T_7_ Rice plants infected with *M. oryzae*. Data are shown as mean ± SE (n = 3) at *p* < 0.05. One-way ANOVA followed by Tukey’s post-hoc test to compare means. ^#^ *p* < 0.001, compared to control group; * *p* < 0.001 compared to disease control group.

**Table 7:** Estimation of total phenol in different nanoparticle treatments of rice plant, infected with blast.

Treatment	Total Phenol (g% of Dry Weight)	% Variation Compared with T_1_
Control	3.32	-
T_1_: 0 ppm AgNPs (DC)	4.29 ^#^	-
T_2_: 05 ppm AgNPs	4.58	6.76
T_3_: 10 ppm AgNPs	4.89 *	13.99
T_4_: 15 ppm AgNPs	5.23 *	21.91
T_5_: 20 ppm AgNPs	5.26 *	22.61
T_6_: 30 ppm AgNPs	5.12 *	19.35
T_7_: 50 ppm AgNPs	6.25 *	45.69
S.E(m)	0.113	
C.D.	0.343	

Control: Rice plants not infected with *M. oryzae.* T_1_–T_7_ Rice plants infected with *M. oryzae*. Data are shown as mean ± SE (n = 3) at *p* < 0.05. One-way ANOVA followed by Tukey’s post-hoc test to compare means. ^#^ *p* < 0.001, compared to control group; * *p* < 0.001 compared to disease control group.

**Table 8:** Diameter of zone of inhibition (mm) observed for green synthesized AgNPs against MDR pathogens.

	Bacteria	Diameter of Inhibition Zone (mm)
		**AgNPs**
1.	*ATCC 25922 E. coli*	12
2.	*E. coli Ec1*	11
3.	*E. coli Ec2*	12
4.	*E. coli Ec3*	11
5.	*E. coli Ec4*	12
6.	*E. coli Ec5*	12
7	*E. coli Ec6*	13
8.	*K. pneumoniae KP-1*	11
9.	*K. pneumoniae KP-2*	11
10.	*K. pneumoniae KP-3*	13
11.	*K. pneumoniae KP-4*	12
12.	*K. pneumoniae KP-5*	12
13.	*K. pneumoniae KP-6*	13
14.	*K. pneumoniae KP-7*	12

**Table 9:** Details of the treatment groups used in the study.

Treatment	Details
Control	0 ppm AgNPs + no disease (Normal control)
T1	0 ppm AgNPs + diseased (Disease control-DC)
T2	05 ppm AgNPs + diseased
T3	10 ppm AgNPs + diseased
T4	15 ppm AgNPs + diseased
T5	20 ppm AgNPs + diseased
T6	30 ppm AgNPs + diseased
T7	50 ppm AgNPs + diseased

## Data Availability

All the data has been presented in this article.

## Sample Availability

Samples of the compounds will be available from the authors.
